# Long-Term Trends in Postoperative Opioid Prescribing, 1994 to 2014

**DOI:** 10.5435/JAAOSGlobal-D-19-00171

**Published:** 2020-01-07

**Authors:** Catherine L. Chen, Molly Moore Jeffery, Erin E. Krebs, Cornelius A. Thiels, Mark A. Schumacher, Adam J. Schwartz, Robert Thombley, Emily Finlayson, Rosa Rodriguez-Monguio, Derek Ward, R. Adams Dudley

**Affiliations:** From the Philip R. Lee Institute for Health Policy Studies at the University of California, San Francisco (Dr. Chen, Dr. Finlayson, Dr. Rodriguez-Monguio, Dr. Ward, and Dr. Dudley), San Francisco, CA; the Center for Healthcare Value, University of California, San Francisco (Dr. Chen), San Francisco, CA; the Department of Anesthesia and Perioperative Care, University of California, San Francisco (Dr. Chen and Dr. Schumacher), San Francisco, CA; OptumLabs Visiting Fellow (Dr. Chen), San Francisco, CA; the Department of Health Sciences Research, Mayo Clinic (Dr. Jeffery), Rochester, MN; the Department of Medicine, University of Minnesota Medical School (Dr. Krebs), Minneapolis, MN; the Center for Care Delivery and Outcomes Research, Minneapolis VA Health Care System (Dr. Krebs), Minneapolis, MN; the Department of Surgery, Memorial Sloan Kettering Cancer Center (Dr. Thiels), New York, NY; the Department of Orthopaedic Surgery, Mayo Clinic (Dr. Schwartz) Phoenix, AZ; the Center for Clinical Informatics and Improvement Research, University of California, San Francisco (Thombley), San Francisco, CA; the Department of Surgery, University of California, San Francisco (Dr. Finlayson), San Francisco, CA; the Department of Clinical Pharmacy, University of California, San Francisco (Dr. Rodriguez-Monguio), San Francisco, CA; the Medication Outcomes Center, University of California, San Francisco (Dr. Rodriguez-Monguio), San Francisco, CA; the Department of Orthopaedic Surgery, University of California, San Francisco (Dr. Ward), San Francisco, CA; and the Department of Medicine, University of California, San Francisco (Dr. Dudley), San Francisco, CA.

## Abstract

Opioids are routinely prescribed to manage acute postoperative pain, but changes in postoperative opioid prescribing associated with the marketing of long-acting opioids such as OxyContin have not been described in the surgical cohort.

**Methods:**

Using a large commercial claims data set, we studied postoperative opioid prescribing after selected common surgical procedures between 1994 and 2014. For each procedure and year, we calculated the mean postoperative morphine milligram equivalents (MME) filled on the index prescription and assessed the proportion of patients who filled a high-dose prescription (≥350 MME). We reported changes in postoperative opioid prescribing over time and identified predictors of filling a high-dose postoperative opioid prescription.

**Results:**

We identified 1,321,264 adult patients undergoing selected common surgical procedures between 1994 and 2014, of whom 80.3% filled a postoperative opioid prescription. One in five surgery patients filled a high-dose postoperative opioid prescription. Between 1994 and 2014, the mean MME filled increased by 145%, 84%, and 85% for lumbar laminectomy/laminotomy, total knee arthroplasty, and total hip arthroplasty, respectively. The procedures most likely to be associated with a high-dose opioid fill were all orthopaedic procedures (AOR 5.20 to 7.55, *P* < 0.001 for all). Patients whose postoperative opioid prescription included a long-acting formulation had the highest odds of filling a prescription that exceeded 350 MME (AOR 32.01, 95% CI, 30.23–33.90).

**Discussion:**

After the US introduction of long-acting opioids such as OxyContin, postoperative opioid prescribing in commercially insured patients increased in parallel with broader US opioid-prescribing trends, most notably among patients undergoing orthopaedic surgical procedures. The increase in the mean annual MME filled starting in the late 1990s was driven in part by the higher proportion of long-acting opioid formulations on the index postoperative opioid prescription filled by orthopaedic surgery patients.

Prescriptions for opioids have increased 10-fold since 1990, and the United States has the highest rate of prescription opioid use per capita in the world, accounting for 80% of the world's opioid consumption.^1,2^ Increased opioid prescriptions were accompanied by an increase in the incidence of opioid abuse, addiction, and overdose.^3,4^ Although prescribing volume peaked in 2010, opioid use continues to be a major public health concern in the United States.^4,5^

Physicians routinely prescribe opioids to patients to manage acute postoperative pain. There are an estimated 100 million operations done per year in the United States, with approximately 60% of those done in an ambulatory setting.^6^ Ninety-nine percent of patients who are hospitalized after undergoing a surgical procedure receive opioids,^7^ and 80% to 90% of patients undergoing outpatient surgery are sent home with an opioid prescription.^8,9^ Both the proportion of patients receiving an opioid prescription and the total amount prescribed after low-risk outpatient surgery have increased since 2004.^8^ However, little is known about longer term patterns of postoperative opioid prescribing. In particular, opioid-prescribing changes associated with the approval and aggressive marketing of long-acting opioids such as OxyContin starting in the mid-1990s and the implementation of the Joint Commission's new hospital pain management standards in the early 2000s have not been described in the surgical cohort.^10,11^ The destigmatization of opioid use and the widespread recognition of pain as a “fifth vital sign” that followed are credited with increasing physician acceptance of opioid prescribing in the United States during that time.^12^

We hypothesized that the total amount of opioids prescribed after surgery increased over time in parallel to the broader contextual changes in opioid prescribing in the United States. Using claims and prescribing data from a cohort of commercially insured patients, we studied postoperative opioid-prescribing patterns after selected common surgical procedures from 1994 to 2014. For each procedure type and year, we calculated the mean oral morphine milligram equivalents (MME) filled after surgery. We reported changes in overall patterns of postoperative opioid prescribing during the 21-year study period and identified predictors of filling a high-dose postoperative opioid prescription.

## Methods

### Study Oversight and Data Source

We studied a cohort of commercially insured enrollees from the OptumLabs Data Warehouse, a longitudinal, real-world data asset with more than 200 million deidentified lives across claims and clinical information.^13^ The claims data set includes inpatient and outpatient claims as well as pharmacy claims dating from 1993 to the present. Because we used deidentified administrative data, this study did not require institutional review board approval or waiver.

### Study Cohort

Using Current Procedural Terminology (CPT) and *International Classification of Diseases, Ninth Revision* (*ICD-9*) diagnosis and procedure codes, we identified patients aged 18 years and older who underwent selected common surgical procedures between 1994 and 2014 and had at least 365 days of continuous enrollment before and 30 days of continuous enrollment after the date of surgery. These procedures were selected because they are common and because procedure-specific CPT and *ICD-9* codes were available throughout the 21-year study period. Table S1 in the Online Supplement, http://links.lww.com/JG9/A60, lists the CPT/*ICD-9* codes used to define our cohort. Patients with claims for two related procedures on the same day (eg, laparoscopic versus open cholecystectomy) were categorized with the more invasive procedure (ie, open cholecystectomy). Patients who underwent unrelated procedures from our list on the same day were excluded. We also excluded anyone with a hospital length of stay (LOS) over 14 days to account for unanticipated complications that could alter a patient's postoperative pain management needs. We extracted additional variables including pre-existing comorbidities, previous prescriptions, and surgery location for our analysis.

### Predictor Variables

We tested the association between postoperative dose prescribed and patient, procedural, and geographic factors, as well as the year the surgery was done. Patient variables included patient demographics such as age, sex, and race; Deyo-Charlson Comorbidity Index; having pre-existing diagnoses associated with opioid use (chronic pain, anxiety, depression, psychosis, post-traumatic stress disorder, alcohol use, tobacco use, or substance abuse) or a current cancer diagnosis (Table S2, http://links.lww.com/JG9/A60); and previous pharmacy claims for medications including opioids, benzodiazepines, muscle relaxants, antidepressants, or antipsychotics. Diagnoses were considered if they were coded within 12 months of the date of surgery. To avoid overlap with the initial postoperative opioid prescription, all preoperative prescription claims (including all preoperative opioid prescriptions) were assessed starting from 12 months up to 30 days before the date of surgery. Procedure-related variables included procedure type, place of service, and hospital LOS for inpatient procedures. Outpatient procedures were designated as having a LOS of zero days.

### Outcome Variable

For each patient, we identified pharmacy claims for the index postoperative opioid prescription fill. Surgeons frequently provide opioid prescriptions to their patients ahead of scheduled surgery. Therefore, we defined the index postoperative opioid prescription fill as the first opioid prescription filled during a 14-day window comprising the 7 days before and after the date of surgery (outpatient procedures) or the date of discharge (inpatient procedures). Using Centers for Disease Control and Prevention (CDC) conversion factors (Table S3, http://links.lww.com/JG9/A60),^14^ we converted each unique opioid, dosage, and duration on the index prescription to its oral morphine equivalent dose in milligrams (MME). If patients filled more than one opioid on the date of the index prescription fill, the MME for each opioid was summed to calculate a total MME for the index prescription.

There are currently no nationally accepted guidelines for postoperative opioid prescribing. In this context, some investigators have used 200 MME as a threshold for excessive postoperative opioid prescribing.^15^ Other existing state and institutional guidelines recommend prescribing a maximum of 150 to 315 MME (∼20 to 42 tablets of oxycodone 5 mg) for most procedures included in our study.^16–18^ Given these guidelines, we adopted a total prescription dosage of ≥350 as a conservative definition of a high-dose postoperative opioid prescription fill.

### Statistical Analysis

We evaluated prescriptions filled from 1994 to 2014. For each procedure and year, we calculated the mean and median postoperative MME filled. To account for the effect of the approval and marketing of long-acting opioids on opioid prescribing,^10^ we identified the proportion of patients whose index postoperative opioid prescription included a long-acting formulation and the proportion who had filled any preoperative prescription for long-acting opioids. We depicted changes in opioid selection over time. We used multiple logistic regression to assess the association of patient, surgery and geographic factors, year of surgery, and inclusion of long-acting opioids with the receipt of ≥350 MME on the index prescription. Wald chi-square tests were used to determine the statistical significance of these predictors.

### Sensitivity Analysis

As a sensitivity analysis, we repeated our analyses in opioid-naive patients (patients without an opioid prescription fill starting from 12 months to 30 days before surgery).

All statistical analyses were done using R version 3.4.3 (The R Foundation).

## Results

We identified 1,321,264 patients meeting inclusion criteria, of whom 1,061,104 (80.3%) filled an index postoperative opioid prescription (Table 1). The mean age was 48.4 years (SD 12.9). Patients were predominantly female (58.9%), white (75.2%), and from the South (45.0%). Approximately 35.3% of patients received preoperative opioids, and 1.7% used long-acting opioids preoperatively. The mean and median index postoperative opioid prescription fill was 268.1 and 200 MME, respectively. Approximately 20.3% of patients received high-dose postoperative opioids (≥350 MME), and 1.8% filled a long-acting postoperative opioid prescription.

**Table 1:** Baseline Characteristics for Patients Undergoing Selected Surgeries, 1994 to 2014

	All Patients 1994-2014, N = 1,321,264	%
Patient characteristics		
Age in years (SD)	48.4 (12.9)	
Sex		
Male	542,423	41.1
Female	778,841	58.9
Race		
White	994,180	75.2
Black	86,679	6.6
Asian	22,394	1.7
Hispanic	91,217	6.9
Unknown	126,794	9.6
Geographic region		
South	594,124	45.0
West	178,110	13.5
Midwest	438,675	33.2
Northeast	110,355	8.4
Deyo-Charlson Comorbidity Index		
0-1	1,130,752	85.6
2	117,167	8.9
3+	73,345	5.6
Active cancer diagnosis	14,518	1.1
Predisposing factors		
Post-traumatic stress disorder	4,694	0.4
Anxiety	101,287	7.7
Depression	143,673	10.9
Psychosis	5,038	0.4
Chronic pain	16,249	1.2
Substance abuse	10,054	0.8
Tobacco use	102,207	7.7
Alcohol use	13,285	1.0
Prescriptions in previous 12 mo		
Any opioid	466,767	35.3
Long-acting opioids	22,877	1.7
Benzodiazepines	176,109	13.3
Muscle relaxants	152,208	11.5
Antidepressants	276,431	20.9
Antipsychotics	6,706	0.5
Surgical characteristics		
Procedure type		
Arthroscopic knee meniscectomy	242,605	18.4
Laparoscopic cholecystectomy	222,762	16.9
Open hysterectomy	106,716	8.1
Open inguinal hernia repair	101,756	7.7
Carpal tunnel release	83,499	6.3
Breast lumpectomy	73,967	5.6
Laparoscopic appendectomy	69,773	5.3
Total knee arthroplasty	68,333	5.2
Lumbar laminectomy/laminotomy	55,175	4.2
Total hip arthroplasty	46,094	3.5
Lap-assisted vaginal hysterectomy	41,705	3.2
Vaginal hysterectomy	38,051	2.9
Open rotator cuff repair	34,610	2.6
Open partial colectomy	33,600	2.5
Laparoscopic inguinal hernia repair	29,615	2.2
Arthroscopic ACL repair	25,968	2.0
Open appendectomy	18,646	1.4
Simple mastectomy	16,232	1.2
Open cholecystectomy	12,157	0.9
Surgery location		
Hospital outpatient department	612,675	46.4
Inpatient hospital	493,900	37.4
Ambulatory surgery center	207,922	15.7
Office	3,776	0.3
Unknown	2,991	0.2
LOS in days		
0	854,627	64.7
1	78,862	6.0
2	137,068	10.4
3+	250,707	19.0
Year of surgery		
1994	8,149	0.6
1995	9,336	0.7
1996	11,094	0.8
1997	13,894	1.1
1998	20,177	1.5
1999	29,511	2.2
2000	37,265	2.8
2001	40,660	3.1
2002	69,661	5.3
2003	84,413	6.4
2004	88,696	6.7
2005	88,027	6.7
2006	101,000	7.6
2007	100,491	7.6
2008	104,517	7.9
2009	99,772	7.6
2010	92,330	7.0
2011	88,620	6.7
2012	85,321	6.5
2013	80,042	6.1
2014	68,288	5.2
Index prescription characteristics		
Any opioid prescription	1,061,104	80.3
High-dose opioid prescription (≥350 MME)	267,630	20.3
Any long-acting opioid prescription	23,401	1.8
Mean postop MME prescribed (SD)	268.1 (515.6)	—
Median postop MME prescribed	200	—
Opioid class prescribed^a^		
Hydrocodone_SA	528,446	49.8
Oxycodone_SA	381,787	36.0
Propoxyphene	81,072	7.6
Codeine	35,083	3.3
Tramadol_SA	20,635	1.9
Oxycodone_LA	18,543	1.7
Meperidine	13,386	1.3
Hydromorphone_SA	10,971	1.0
Morphine_LA	2,475	0.2
Tapentadol_SA	1,561	0.1
Fentanyl_LA	1,457	0.1
Pentazocine	1,247	0.1
Morphine_SA	712	0.1
Other	1,549	0.1

ACL = anterior cruciate ligament, LA = long-acting, LOS = length of stay, MME = oral morphine milligram equivalents, SA = short-acting

a Percentages represent proportion of all opioids prescribed.

**Figure 1:**
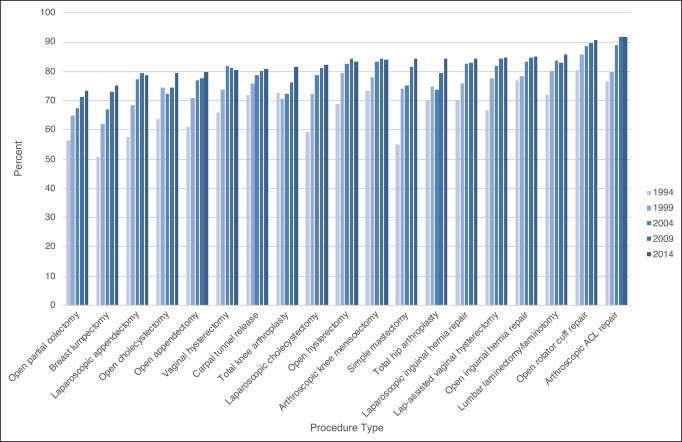
Percent of patients who filled an index postoperative opioid prescription, 1994 to 2014.

**Figure 2:**
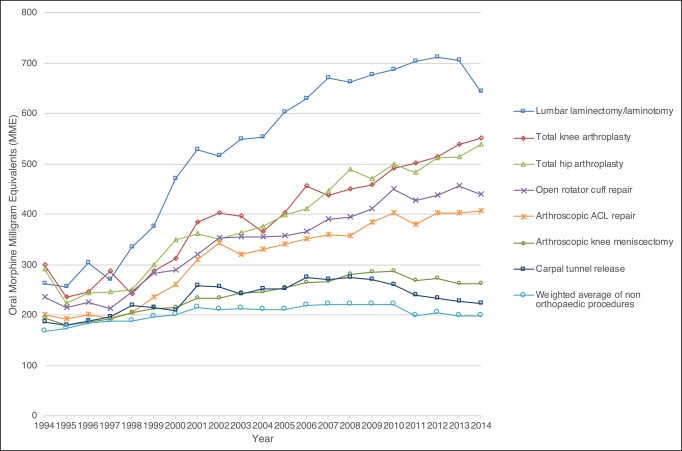
Mean oral morphine equivalents prescribed on index postoperative opioid prescription fill, 1994 to 2014.

**Figure 3:**
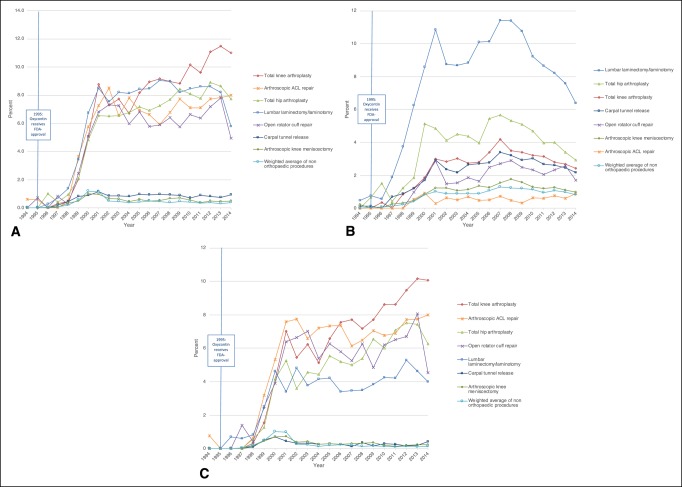
**A**, Percent of patients with any long-acting opioid on index postoperative opioid prescription fill, 1994 to 2014. Denominator includes all patients in cohort, not just patients who filled an index postoperative opioid prescription. **B**, Percent of patients who filled any long-acting opioid prescription in the 12 months preceding surgery, 1994 to 2014. Preoperative long-acting opioid prescription claims were assessed starting from 12 months up to 30 days before the date of surgery. **C**, Percent of patients with any long-acting opioid on index postoperative opioid prescription fill, opioid-naive patients only, 1994 to 2014. Denominator includes all opioid-naive patients in cohort, not just patients who filled an index postoperative opioid prescription.

The prevalence of postoperative opioid prescription fills increased from 67.7% of patients in 1994 to 82.8% in 2014 and was generally higher among patients undergoing orthopaedic procedures (Figure 1). The 2014 prevalence was highest for patients undergoing arthroscopic anterior cruciate ligament (ACL) repair (91.9%), open rotator cuff repair (90.6%), and lumbar laminectomy/laminotomy (85.8%). The mean and median postoperative MME filled each year for each procedure type also increased over time, particularly among patients undergoing orthopaedic procedures (Figure 2, Figures S1 and S2, http://links.lww.com/JG9/A60). Between 1994 and 2014, the mean postoperative MME filled increased by 145% for lumbar laminectomy/laminotomy, 84% for total knee arthroplasty, and 85% for total hip arthroplasty. All procedures showed a spike in prevalence of long-acting prescription fills in the year 2000 (prevalence ranged from 0.6% for breast lumpectomy to 6.7% for lumbar laminectomy/laminotomy); however, we found that the prescriptions filled by patients undergoing orthopaedic procedures had a sustained increase in the use of long-acting opioids (Figure 3A; Figure S3, http://links.lww.com/JG9/A60). By 2012, 11.1% of patients undergoing total knee arthroplasty, 8.9% undergoing total hip arthroplasty, and 8.6% undergoing lumbar laminectomy/laminotomy filled a long-acting opioid prescription. Preoperative use of long-acting opioids increased substantially during the study period, especially among patients undergoing lumbar laminectomy/laminotomy (Figure 3B; Figure S4, http://links.lww.com/JG9/A60). We found that patients who had previously been opioid-naive were also filling long-acting opioid prescriptions postoperatively (Figure 3C; Figure S5, http://links.lww.com/JG9/A60). Additional longitudinal trends in the opioid-naive cohort were consistent with our main findings (Figures S6 to S8, http://links.lww.com/JG9/A60).

Prescribing of specific opioid medications changed over time (Figure S9, http://links.lww.com/JG9/A60). When looking at all procedures combined, hydrocodone and oxycodone were used in approximately 60% of postoperative opioid prescriptions filled in 1994, compared with 92% in 2014. The proportion of prescription fills that included codeine declined from 19% in 1994 to 3% in 2014. Propoxyphene declined from 21% in 1994 to 0% in 2011 after it was removed from the market in 2010 because of its association with fatal cardiac arrhythmias.^19^ After FDA approval in 1995, tramadol use increased to 5% in 2014, while long-acting oxycodone use increased to 2% in 2014. Similar changes in prescribing occurred in the opioid-naive cohort (Figure S10, http://links.lww.com/JG9/A60).

The likelihood of filling a high-dose postoperative opioid prescription was associated with patient-related variables such as younger age, white race, and the presence of predisposing diagnoses such as chronic pain Adjusted odds ratio (AOR) 1.35, 95% Confidence Interval (CI): 1.31, 1.40), substance abuse (AOR 1.07; 95% CI, 1.02, 1.13), and tobacco use (AOR 1.07; 95% CI, 1.05, 1.09) (Table 2). Prescription drug use in the 12 months before surgery was also associated with a high-dose fill, including antidepressants (AOR 1.11, 95% CI, 1.10, 1.13), muscle relaxants (AOR 1.15, 95% CI, 1.13, 1.16), benzodiazepines (AOR 1.17, 95% CI, 1.15, 1.18), any opioid (AOR 1.27, 95% CI, 1.25, 1.28), and any long-acting opioid (AOR 1.30, 95% CI, 1.26, 1.35).

**Table 2:** Predictors of Receiving High-Dose Opioids on Index Postoperative Opioid Prescription

	Unadj. OR	(95% CI)	Adj. OR	(95% CI)	*P* (Wald test)
Age (yr)					
18-35 (ref)	—	—	—	—	—
35-45	1.10	(1.09, 1.12)	1.01	(0.99, 1.03)	0.187
45-55	1.34	(1.32, 1.36)	0.99	(0.98, 1.01)	0.255
55-65	1.67	(1.65, 1.69)	0.92	(0.90, 0.93)	<0.001
65-75	1.46	(1.43, 1.49)	0.67	(0.66, 0.69)	<0.001
75+	0.97	(0.93, 1.00)	0.38	(0.37, 0.40)	<0.001
Sex					
Male (ref)	—	—	—	—	—
Female	0.87	(0.86, 0.88)	1.00	(0.99, 1.02)	0.479
Race					
White (ref)	—	—	—	—	—
Black	0.90	(0.89, 0.92)	0.91	(0.90, 0.93)	<0.001
Asian	0.74	(0.71, 0.77)	0.87	(0.83, 0.90)	<0.001
Hispanic	0.79	(0.77, 0.80)	0.95	(0.93, 0.97)	<0.001
Unknown	0.78	(0.77, 0.79)	0.96	(0.94, 0.98)	<0.001
Geographic region					
South (ref)	—	—	—	—	—
West	1.05	(1.04, 1.07)	0.95	(0.94, 0.97)	<0.001
Midwest	0.96	(0.95, 0.97)	0.94	(0.93, 0.96)	<0.001
Northeast	0.71	(0.69, 0.72)	0.69	(0.68, 0.70)	<0.001
Deyo-Charlson Comorbidity Index					
0-1 (ref)	—	—	—	—	—
2	1.04	(1.03, 1.06)	0.97	(0.96, 0.99)	0.003
3+	1.12	(1.10, 1.14)	1.00	(0.98, 1.03)	0.678
Active cancer diagnosis	1.01	(0.97, 1.05)	0.97	(0.92, 1.01)	0.169
Predisposing factors^a^					
Post-traumatic stress disorder	1.31	(1.22, 1.40)	0.96	(0.89, 1.04)	0.328
Anxiety	1.20	(1.19, 1.22)	0.99	(0.97, 1.01)	0.273
Chronic pain	3.28	(3.18, 3.38)	1.35	(1.30, 1.41)	<0.001
Substance abuse	1.91	(1.84, 2.00)	1.07	(1.02, 1.13)	0.008
Tobacco use	1.30	(1.28, 1.32)	1.07	(1.05, 1.09)	<0.001
Alcohol use	1.32	(1.27, 1.38)	0.97	(0.93, 1.02)	0.268
Prescriptions in previous 12 mo					
Any opioid	1.89	(1.87, 1.91)	1.27	(1.25, 1.28)	<0.001
Long-acting opioids	4.24	(4.13, 4.35)	1.30	(1.26, 1.35)	<0.001
Benzodiazepines	1.53	(1.51, 1.54)	1.17	(1.15, 1.18)	<0.001
Muscle relaxants	1.84	(1.82, 1.86)	1.15	(1.13, 1.16)	<0.001
Antidepressants	1.38	(1.37, 1.40)	1.11	(1.10, 1.13)	<0.001
Antipsychotics	1.72	(1.63, 1.81)	1.04	(0.98, 1.10)	0.220
Procedure type					
Laparoscopic cholecystectomy (ref)	—	—	—	—	—
Lumbar laminectomy/laminotomy	10.20	(9.98, 10.42)	7.55	(7.37, 7.74)	<0.001
Total hip arthroplasty	8.07	(7.89, 8.25)	6.72	(6.54, 6.91)	<0.001
Total knee arthroplasty	7.80	(7.65, 7.96)	6.64	(6.47, 6.81)	<0.001
Arthroscopic ACL repair	5.61	(5.45, 5.77)	5.44	(5.27, 5.60)	<0.001
Open rotator cuff repair	5.33	(5.20, 5.46)	5.20	(5.06, 5.35)	<0.001
Simple mastectomy	2.00	(1.92, 2.08)	1.78	(1.70, 1.86)	<0.001
Arthroscopic knee meniscectomy	1.88	(1.85, 1.91)	2.10	(2.06, 2.14)	<0.001
Open cholecystectomy	1.81	(1.73, 1.90)	1.72	(1.63, 1.81)	<0.001
Carpal tunnel release	1.58	(1.54, 1.62)	1.67	(1.63, 1.71)	<0.001
Open partial colectomy	1.53	(1.48, 1.58)	1.50	(1.45, 1.56)	<0.001
Open hysterectomy	1.38	(1.35, 1.41)	1.20	(1.17, 1.23)	<0.001
Breast lumpectomy	1.25	(1.22, 1.28)	1.50	(1.46, 1.54)	<0.001
Lap-assisted vaginal hysterectomy	1.16	(1.12, 1.20)	1.02	(0.99, 1.05)	0.288
Vaginal hysterectomy	1.12	(1.08, 1.16)	1.02	(0.98, 1.05)	0.301
Open inguinal hernia repair	1.01	(0.98, 1.03)	1.21	(1.18, 1.24)	<0.001
Open appendectomy	0.98	(0.93, 1.03)	1.01	(0.96, 1.06)	0.835
Laparoscopic inguinal hernia repair	0.85	(0.82, 0.89)	0.96	(0.92, 1.00)	0.041
Laparoscopic appendectomy	0.75	(0.73, 0.77)	0.73	(0.71, 0.75)	<0.001
Surgery location					
Hospital outpatient department (ref)	—	—	—	—	—
Inpatient hospital	1.82	(1.80, 1.84)	0.91	(0.89, 0.94)	<0.001
Ambulatory surgery center	1.19	(1.17, 1.20)	0.93	(0.92, 0.94)	<0.001
Office	0.88	(0.81, 0.97)	0.82	(0.75, 0.90)	<0.001
Unknown	1.22	(1.12, 1.34)	0.90	(0.81, 0.99)	0.033
LOS					
0 (ref)	—	—	—	—	—
1	1.19	(1.17, 1.21)	1.23	(1.20, 1.27)	<0.001
2	1.61	(1.59, 1.63)	1.48	(1.44, 1.53)	<0.001
3+	2.08	(2.06, 2.10)	1.27	(1.23, 1.30)	<0.001
Postop prescription included LA opioid	70.94	(67.12, 74.99)	32.01	(30.23, 33.90)	<0.001
Year of surgery					
1994 (ref)	—	—	—	—	—
1995	0.98	(0.90, 1.07)	0.98	(0.89, 1.07)	0.617
1996	1.04	(0.96, 1.13)	1.01	(0.93, 1.10)	0.742
1997	1.07	(0.99, 1.16)	1.03	(0.95, 1.12)	0.432
1998	1.06	(0.99, 1.14)	1.00	(0.92, 1.08)	0.944
1999	1.14	(1.07, 1.23)	1.03	(0.96, 1.11)	0.444
2000	1.23	(1.15, 1.31)	1.05	(0.98, 1.13)	0.161
2001	1.34	(1.26, 1.44)	1.13	(1.06, 1.22)	<0.001
2002	1.25	(1.17, 1.34)	1.03	(0.96, 1.11)	0.349
2003	1.33	(1.24, 1.41)	1.07	(1.00, 1.15)	0.052
2004	1.33	(1.25, 1.42)	1.07	(1.00, 1.14)	0.059
2005	1.39	(1.30, 1.48)	1.09	(1.02, 1.17)	0.011
2006	1.54	(1.45, 1.65)	1.21	(1.13, 1.30)	<0.001
2007	1.60	(1.50, 1.71)	1.23	(1.15, 1.32)	<0.001
2008	1.73	(1.62, 1.84)	1.32	(1.23, 1.41)	<0.001
2009	1.79	(1.68, 1.91)	1.34	(1.26, 1.44)	<0.001
2010	1.85	(1.74, 1.98)	1.39	(1.30, 1.49)	<0.001
2011	1.62	(1.52, 1.73)	1.17	(1.10, 1.26)	<0.001
2012	1.73	(1.62, 1.85)	1.25	(1.17, 1.34)	<0.001
2013	1.80	(1.68, 1.92)	1.28	(1.19, 1.37)	<0.001
2014	1.90	(1.78, 2.02)	1.37	(1.27, 1.46)	<0.001

ACL = anterior cruciate ligament, LA = long-acting, LOS = length of stay, MME = oral morphine milligram equivalents, OR = odds ratio

a Depression was removed as predictor variable because it was highly colinear with antidepressant use in the previous 12 months.

Patients who were discharged on postoperative day #2 had higher adjusted odds of filling a high-dose postoperative opioid prescription (1.48, 95% CI, 1.44, 1.53), compared with patients with shorter or longer hospitalizations or having ambulatory surgery. Year of surgery from the early 2000s through 2014 was also associated with higher adjusted odds of having a high-dose postoperative prescription fill. The procedures with the highest adjusted odds of patients filling a high-dose postoperative opioid prescription were all orthopaedic procedures: lumbar laminectomy/laminotomy (7.55, 95% CI, 7.37, 7.74), total hip arthroplasty (6.72, 95% CI, 6.54, 6.91), total knee arthroplasty (6.64, 95% CI, 6.47, 6.81), arthroscopic anterior cruciate ligament repair (5.44, 95% CI, 5.27, 5.60), and open rotator cuff repair (5.20, 95% CI, 5.06, 5.35). Patients whose postoperative opioid prescription included a long-acting formulation had the highest adjusted odds of filling a high-dose prescription (AOR 32.01, 95% CI, 30.23, 33.90).

In a sensitivity analysis limited to opioid-naive patients (Table S4, http://links.lww.com/JG9/A60), procedure type and use of a long-acting formulation in the postoperative prescription remained the strongest predictors for filling a high-dose postoperative opioid prescription (Table S5, http://links.lww.com/JG9/A60).

## Discussion

In this study of long-term postoperative opioid-prescribing trends in commercially insured patients, we found that changes in the characteristics of the index postoperative opioid prescription filled by adult patients undergoing common surgical procedures mirrored the broader increase in opioid prescribing in the United States.^1,2^ Postoperative opioid prescription fills increased in both prevalence and total MME prescribed, and—after new long-acting opioids such as OxyContin became widely marketed—in the likelihood that a long-acting formulation was included.

The increase in the prevalence and dose of postoperative opioids is notable because it occurred despite improvements in both surgical technique and perioperative pain management approaches during the same period. The use of minimally invasive approaches, advances in neuraxial and regional anesthetic techniques, and the introduction of enhanced recovery pathways and multimodal analgesic approaches for many of these procedures should have decreased patients' postoperative pain and the opioid dosage on the index prescription that would have been expected to meet their needs.^20,21,22,23^ However, these perioperative advances coincided with the introduction and marketing of OxyContin and other long-acting opioids in the mid-1990s,^10,24^ the simultaneous liberalization of what were considered to be appropriate indications for receiving a new opioid prescription,^25^ and the implementation of the Joint Commission standards for the evaluation and treatment of pain among hospitalized patients in the early 2000s.^12,26^ Others have noted that the increased likelihood of preexisting opioid tolerance and the trend toward same-day surgeries and earlier discharge after inpatient surgery also may have contributed to escalating opioid dosages, compared with an earlier epoch when more patients would have been opioid-naive before surgery and would have had longer inpatient stays after surgery.^1,12,26^ In addition, pharmaceutical manufacturers may have targeted marketing to certain physicians preferentially based on their specialty.^10,20,24,27^

These broader influences on postoperative opioid prescribing are illustrated by our study results, particularly among patients undergoing orthopaedic surgery. The strongest predictor of receiving a high-dose opioid prescription in our study was whether the prescription included a long-acting opioid, followed closely by whether patients were undergoing one of several common orthopaedic procedures. Our study does not specifically address the factors that may have led to the more frequent reliance on long-acting opioids in orthopaedic surgery patients postoperatively compared with patients undergoing nonorthopaedic procedures. Notably, most of the orthopaedic procedures included in our study are indicated to treat chronic musculoskeletal pain, which makes it more likely that these patients were already receiving opioids preoperatively. This is evidenced by the longitudinal increase in prevalence of long-acting opioids, especially among patients undergoing lumbar laminectomy/laminotomy.

However, our results among opioid-naive patients showed similar increases in MMEs prescribed over time. Consistent with our findings in the overall cohort, the inclusion of a long-acting formulation and undergoing the same orthopaedic procedures were the strongest predictors of filling a high-dose prescription in the opioid-naive cohort as well. Others have reported on the inclusion of OxyContin in orthopaedic clinical pathways to facilitate earlier hospital discharge, which may be contributing to the greater prescription dosages in these patients.^20,27^ These results suggest that postoperative opioid-prescribing practice changes over time were not driven solely by patients' preoperative opioid use.

The use of OxyContin in the postoperative setting continued despite changes in the labeling of OxyContin in July 2001 that specified it should not be used to treat pain in the immediate postoperative period.^28^ Others have shown that the initiation of postoperative opioids in previously opioid-naive patients is associated with increased risk of chronic use,^29,30^ and introducing long-acting opioids to opioid-naive patients is associated with increased all-cause mortality.^31^ For many patients, surgery is the instigating event for first opioid use; therefore, surgeons have a particular responsibility to avoid the inappropriate use of long-acting opioids.^32,33^

Our study has several limitations. First, there were changes to the OptumLabs data set over the 21-year study period, including changes in number of covered lives and case mix represented. Second, we studied selected surgical procedures in a commercially insured cohort, which may limit the generalizability of our findings to patients undergoing other procedures or who are not commercially insured. Other limitations related to the use of administrative claims data also apply. Third, some of the index prescriptions filled within our designated 14-day prescribing window may have been provided for indications unrelated to surgery. Furthermore, the prescriptions we analyzed only included those that were filled and submitted to insurance. Therefore, the exclusion of written but unfilled opioid prescriptions could have affected our results, since patients who fill opioid prescriptions after surgery may differ from patients who receive an opioid prescription but do not fill it. Fourth, the goal of this study was to understand long-term postoperative opioid-prescribing trends reflected by the index postoperative opioid prescription during the years immediately after the introduction of new long-acting opioids such as OxyContin through the peak of the prescription opioid epidemic; postoperative opioid-prescribing practices may have changed since 2014. Finally, our high-dose prescription threshold of 350 MME may not be the optimal threshold for all types of surgical pain, especially for more invasive procedures such as total knee arthroplasty. However, since there are currently no nationally accepted standards for postoperative opioid prescribing in the surgical cohort, our designation of 350 MME as a threshold for high-dose prescribing provides a starting point for further discussion.

In summary, we found that postoperative opioid prescribing in commercially insured patients increased in parallel with broader US opioid-prescribing trends since the mid-1990s, especially among patients undergoing orthopaedic surgical procedures. The increase in the mean annual MME filled starting in the late 1990s was driven in part by the higher proportion of long-acting opioid formulations on the index postoperative opioid prescription filled by orthopaedic surgery patients. Understanding the factors that contribute to high-dose opioid prescribing after surgery is critical to maximizing the benefits of opioid therapy while avoiding opioid-associated adverse events. Surgeons should continue to limit the use of long-acting opioids in the immediate postoperative setting as a way to reduce high-dose prescription fills in patients undergoing orthopaedic surgical procedures.
